# Neonatal *AVPR1a* Methylation and In-Utero Exposure to Maternal Smoking

**DOI:** 10.3390/toxics11100855

**Published:** 2023-10-13

**Authors:** Nichole Nidey, Katherine Bowers, Lili Ding, Hong Ji, Robert T. Ammerman, Kimberly Yolton, E. Melinda Mahabee-Gittens, Alonzo T. Folger

**Affiliations:** 1 Department of Epidemiology, University of Iowa College of Public Health, Iowa City, IA 52242, USA; nichole-nidey@uiowa.edu; 2 Division of Biostatistics and Epidemiology, Cincinnati Children’s Hospital Medical Center, Department of Pediatrics, University of Cincinnati College of Medicine, Cincinnati, OH 45229, USA; katherine.bowers@cchmc.org (K.B.); lili.ding@cchmc.org (L.D.); 3 Department of Anatomy, Physiology and Cell Biology, School of Veterinary Medicine, University of California, Davis, CA 95616, USA; hgji@ucdavis.edu; 4 Division of Behavioral Medicine and Clinical Psychology, Cincinnati Children’s Hospital Medical Center, Department of Pediatrics, University of Cincinnati College of Medicine, Cincinnati, OH 45229, USA; robert.ammerman@cchmc.org; 5 Division of General and Community Pediatrics, Cincinnati Children’s Hospital Medical Center, Department of Pediatrics, University of Cincinnati College of Medicine, Cincinnati, OH 45229, USA; kimberly.yolton@cchmc.org; 6 Division of Emergency Medicine, Cincinnati Children’s Hospital Medical Center, Department of Pediatrics, University of Cincinnati College of Medicine, Cincinnati, OH 45229, USA; melinda.mahabee-gittens@cchmc.org

**Keywords:** maternal smoking, epigenetics, *AVPR1a*, DNA methylation

## Abstract

(1) Introduction: Epigenetic changes have been proposed as a biologic link between in-utero exposure to maternal smoking and health outcomes. Therefore, we examined if in-utero exposure to maternal smoking was associated with infant DNA methylation (DNAm) of cytosine-phosphate-guanine dinucleotides (CpG sites) in the arginine vasopressin receptor 1A *AVPR1a* gene. The *AVPR1a* gene encodes a receptor that interacts with the arginine vasopressin hormone and may influence physiological stress regulation, blood pressure, and child development. (2) Methods: Fifty-two infants were included in this cohort study. Multivariable linear models were used to examine the effect of in-utero exposure to maternal smoking on the mean DNAm of CpG sites located at *AVPR1a.* (3) Results: After adjusting the model for substance use, infants with in-utero exposure to maternal smoking had a reduction in DNAm at *AVPR1a* CpG sites by −0.02 (95% CI −0.03, −0.01) at one month of age. In conclusion, in-utero exposure to tobacco smoke can lead to differential patterns of DNAm of *AVPR1a* among infants. Conclusions: Future studies are needed to identify how gene expression in response to early environmental exposures contributes to health outcomes.

## 1. Introduction

Increased morbidity and mortality related to cigarette smoking is well documented in the extant literature. Despite being the number one preventable cause of death and disease in the United States (US), an estimated 47.1 million adults were currently smoking in 2020 and in 2019, and rates of smoking during pregnancy ranged from 1.1% in California to 23.0% in West Virginia [1,2,3,4,5]. The effects of in-utero exposure to nicotine, one of the thousands of chemicals contained in tobacco smoke, have been extensively studied due to its ability to cross the placental barrier [6,7]. Studies have demonstrated that poor fetal and infant growth, low birth weight, and preterm birth are associated with in-utero exposure to tobacco smoke [8,9,10,11]. A recent study observed in-utero exposure to tobacco smoke is associated with a blunted stress response [12]. Additionally, individuals with in-utero exposure are more likely to have high blood pressure than their unexposed peers [13,14,15,16,17]. Despite this evidence, the mechanisms of risk are not well understood. As a result, studies have begun to examine the epigenetic effects of in-utero exposure to nicotine, using an epigenome-wide approach. These studies have identified unique DNA methylation (DNAm) patterns among individuals with in-utero exposure to nicotine [18]. Prior epigenome-wide association studies (EWAS) have been successful in identifying cytosine-phosphate-guanine dinucleotides (CpG sites) and genes of interest [18]. Candidate gene studies can complement the use of epigenome-wide studies by providing a concentrated analysis of regulatory sites within potentially relevant genes. These analyses can help explicate potential epigenetic mechanisms that link environmental exposures to health and developmental outcomes.

Exposure to nicotine is associated with the release of arginine vasopressin (AVP), a neuroendocrine hormone that causes contraction of peripheral blood vessels by interacting with its receptor and arginine vasopressin receptor 1A (*AVPR1a*) [19,20]. Few candidate gene studies have examined if in-utero exposure to tobacco smoke influences DNAm of the *AVPR1a* gene. This gene is located on chromosome 12q14.2 and encodes the receptors for AVP [21,22].

Nicotine and cotinine easily cross the placental barrier and have been shown to maintain higher fetal concentrations than maternal concentrations [23]. Nicotine binds to and activates nicotinic acetylcholine receptors (nACHRs) [24], which are present in individuals as early as four weeks of gestation [25]. As a result, maternal smoking can have physiological effects in the fetus. Generally, AVP functions to maintain homeostasis of the hypothalamic–pituitary (HPA) axis by releasing adrenocorticotropic hormones (ACTH), in turn leading to the stimulation of corticotropin-releasing hormones (CRH), which stimulate the release of cortisol [20,26]. Animal studies have demonstrated that exposure to nicotine can activate the HPA axis by increasing levels of corticosterone and ACTH [27]. In human studies, nicotine is associated with increased cortisol levels [28]. In a recent observational study, it was demonstrated that in-utero exposure to maternal smoking was associated with a blunted stress response, which suggests that in-utero exposure can lead to infant HPA dysregulation [12]. Additionally, studies have posited that in-utero exposure is related to fetal programming of an infant’s HPA axis, potentially leading to increased risk of metabolic health conditions, such as high blood pressure and diabetes mellitus [12,29,30].

Epigenetic changes have been proposed as a biologic link between maternal exposures and offspring outcomes [31,32]. Maternal and infant physiologic changes in response to nicotine exposure have motivated several studies to examine if exposure to tobacco smoking induces epigenetic changes in offspring, specifically DNAm levels. Numerous studies have identified epigenetic changes among individuals who were exposed to maternal smoking during fetal development [33,34,35]. It is postulated that these epigenetic effects are related to an increased risk of poor birth outcomes and long-term health effects [36]. Epigenome-wide studies of DNA methylation in offspring have identified multiple CpG sites associated with maternal smoking [18]. Examination of the epigenetic effects of in-utero exposure to maternal smoking can help elucidate how exposure leads to poor health outcomes observed in previous studies. Expression of *AVPR1a* may function as a mediator in the relationship of maternal smoking and child health due to its important role in maintaining homeostasis in the HPA axis, blood pressure regulation, and child development [20,26,37]. However, *AVPR1a* has not reached genome-wide significance in prior EWAS studies. The objective of this study was to take a targeted gene approach and examine if in-utero exposure to maternal smoking was associated with infant DNAm patterns of CpG sites in *AVPR1a*. Prior work demonstrates that tobacco smoke exposure is associated with hypertension in adults [38] and other work indicates that prenatal tobacco smoke exposure is associated with dysregulation of the HPA axis [30], risk of metabolic syndrome, and [36] overall differential DNAm patterns compared to unexposed infants [39]. Thus, we hypothesized that we would observe differential DNAm patterns between groups.

## 2. Methods

Children born from December 2015 to October 2016, along with their mothers, participating in the Pregnancy and Infant Development (PRIDE) study [40], were included in this longitudinal cohort study. Briefly, this cohort included 53 mother–child dyads who were recruited from Every Child Succeeds, a home visiting program serving women in greater Cincinnati, Ohio. Participants for this pilot wave of the PRIDE study were selected exclusively from Hamilton County, Ohio. Home visitors referred an estimated 30% of eligible mothers (*n* = 75) to the PRIDE study, where 91% (*n* = 68) expressed interest in enrolling in the study. After screening for eligibility, 56 mothers were enrolled and a total of 53 mothers completed both the prenatal and postnatal study visits. Mothers were eligible for the PRIDE study if they were at least 18 years old, spoke English, and were between 12- and 35-weeks of gestation at the time of enrollment.

Maternal demographics were collected in-person on a demographic form and included age, race, education, and income. Smoking, illicit substance use, and pregnancy complications were also obtained via self-report. These items were collected at the postnatal visit, and the question used for the current study was as follows: “At any time during your pregnancy did you smoke cigarettes?” The amount and frequency of cigarette smoking was not collected. This study was reviewed and approved by the Cincinnati Children’s Hospital Medical Center Institutional Review board (IRB#2015-5583) and was performed in accordance with the Declaration of Helsinki. All participants gave informed consent following IRB guidelines.

### 2.1. DNA Methylation

Buccal sample collection and DNAm measurements for this study have been previously described [40]. Briefly, buccal samples from infants at one month of age were collected by trained study staff. A total of 10 samples were collected by swabbing the inner cheek of each infant with a sponge from the DNAGenotek OGR-250 kits (Ottawa, ON, Canada), with five swabs for DNA collection and five to estimate cell type heterogeneity. The Genomics, Epigenomics, and Sequencing Core at the University of Cincinnati assayed each sample for DNA methylation at over 850,000 CpG sites using the Illumina Infinium Methylation Epic Bead Chip (San Diego, CA, USA).

### 2.2. Array Processing and Quality Control

The Methylation Epic Bead Chip microarray was used to measure DNA methylation intensity at approximately 850 000 CpG sites. The raw DNAm data were processed and analyzed with the R package minfi version 1.26.2 [41,42]. Sample-level quality control methods included Illumina controls, kernel density plots of beta values, and predicted sex checks. The array quality was examined using ewastools package version 1.5. At the CpG probe level, sites were excluded if they had a detection *p* value > 0.01, small bead count (<3) in at least 5% of the samples, or located at SNPs with a minor allele frequency ≥5%. Probes that were known to have non-specific cross-hybridization with other regions or were on sex chromosomes were also excluded.

The normal–exponential with out-of-band probes (noob) within-array approach was used to normalize the intensity data and to perform dye bias adjustment [41,43]. The Enmix package was used for probe type I and type II adjustment [44]. The β values (0 = unmethylated, 1 = fully methylated) and M values (logit transformation of methylation percentage) were derived using normalized intensities. The processed methylation data were filtered to include only CpG sites on the microarray that were within the *AVPR1a* gene. Two of the 10 CpG sites associated with the *AVPR1a* gene were excluded during the quality control process, leaving 8 sites for analysis in this study.

### 2.3. Statistical Analysis

We examined maternal and infant characteristics of our study sample using descriptive analyses. Bivariate associations between smoking and maternal and infant characteristics were examined using Pearson and Fisher’s exact and Chi-square tests where appropriate. Using simple and multivariable linear models, we examined the effect of in-utero exposure to maternal smoking on infant DNAm at one month of age. To reduce the multiple testing burden, we averaged the percent of methylation (beta values) of all eight sites included in this study. Next, we transformed the DNAm beta value to an M-Value (log_2_ ratio of methylation percentage), as the statistical properties of M-values are preferred to beta values [45], to use as the main outcome in the statistical models for *p* values. Maternal and infant characteristics potentially associated with smoking (preterm birth, race/ethnicity, breastfed at least one time during delivery hospitalization, and drug use during pregnancy) were explored as potential confounders to include in the adjusted model. Covariates with *p* values of 0.05 or less were retained and not in the potential causal pathway.

In a secondary analysis, the association between maternal smoking and the infant DNA methylation level of each individual CpG site was examined, using the M-values to determine statistical significance and percent methylation (beta values) to aid in the interpretation. The *p* values were corrected for multiple testing using the false discovery rate controlled at 5%. All statistical analyses were completed in SAS 9.4.

## 3. Results

A total of 53 mother–infant dyads were included in this study, with approximately 19% who reported smoking during pregnancy. One dyad was excluded due to sex mismatch. In total, ~100% of the cell types from saliva samples were epithelial cells; therefore, we did not need to correct for cell types in statistical models. Maternal age in our sample ranged from 18 to 34 years old, with a mean maternal age of 22 years old. Almost 98% of the mothers in our study were low income (defined as 200% below federal poverty level) and 96% were insured by Medicaid at the time of delivery. Approximately 33% reported their race as white and 65% black. When examining the association of maternal and infant characteristics with smoking, we observed mothers who smoked during pregnancy were more likely to use illicit substances and give birth preterm and less likely to breastfeed their infants at least one time (Table 1).

The average percent methylation of the eight *AVPR1a* CpG sites included in this study of infants born to mothers who did not smoke was 0.15, and the mean DNAm among infants born to mothers who smoked during pregnancy was slightly lower at 0.13. In a linear model adjusted for maternal substance use, at one month of age, infants with in-utero exposure to maternal smoking had a significant reduction in DNAm at *AVPR1a* CpG sites by −0.02 ((95% CI −0.03, −0.01) *p* < 0.0001). While preterm birth and the lack of breastfeeding were significantly associated with maternal smoking, we did not adjust for these as they are potential mediators.

### Individual CpG Sites DNAm

In the secondary analysis, we examined the association of exposure to maternal smoking in-utero and the DNAm of each CpG site included in this study. Maternal smoking was associated with lower DNAm at CpG12807275, CpG04827692, and CpG09208611. After correcting for multiple testing, all three sites remained significant (FDR < 0.05, Table 2). Using the UCSC Genome Browser, we determined two of these CpG sites are located within the 1st exon (CpG04827692, CpG09208611) and one site is located within the body of *AVPR1a* (CpG12807275) [46].

## 4. Conclusions

The epigenome is remodeled numerous times during developmental events, such as germ cell specification and differentiation. During remodeling events, the epigenome is particularly vulnerable to environmental insults, such as exposure to chemicals like nicotine. Exposures during this critical period of development and remodeling can lead to changes in the epigenome which could potentially result in long-term health consequences. For example, studies have demonstrated that in-utero maternal smoking exposure exerts effects on infant DNAm at birth and can be observed throughout an individual’s lifespan [47,48]. In this study, we examined the relationship between in-utero exposure to maternal smoking and the DNAm of CpG sites within the *AVPR1a* gene. At one month of age, infants with in-utero exposure to maternal smoking had lower mean DNAm of the CpG sites in this study when compared to unexposed infants. This suggests that in-utero exposure to chemicals contained in tobacco cigarettes, such as nicotine, can lead to changes to the epigenome which may manifest as changes in DNAm. Understanding epigenetic changes in response to early life exposures will help elucidate disease processes and extend our understanding of how maternal health and behavior can transmit health risk in their offspring. *AVPR1a* codes for AVP receptor proteins and has been associated with blood pressure regulation in prior studies [49]. Prior studies have demonstrated that in-utero exposure to maternal smoking is associated with higher blood pressure and an increased risk of hypertension from early childhood through adulthood [13,14,15,16]. Furthermore, mouse models have shown exposure to nicotine during fetal development has been found to affect cardiovascular and renal development [50]. In our study, we observed differential DNAm by exposure to in-utero maternal smoking in the average DNAm of CpG sites. When examining individual CpG sites, three remained significant after adjustment for multiple testing (CpG12807275, CpG04827692, and CpG09208611).

Strengths of this study include using a candidate gene approach and longitudinal assessments to examine the effect of in-utero exposure on the DNAm of CpG sites of the *AVPR1a* gene. Our cohort included an at-risk population of low-income women enrolled in a regional home visiting program. Methods used to examine array quality and the transformation of the DNAm beta value to an M-Value are also strengths. However, results should be interpreted with acknowledgement of important limitations, such as self-reported smoking status and lack of data on number of cigarettes smoked or the timing of when smoking occurred during pregnancy. A prior EWAS study of infant DNAm reported smoking intensity and duration influence DNAm [51]; however, due to limitations of the data we collected, we were not able to examine how these factors would contribute to our study’s findings. This study was also limited by a small sample size, and we did not have adequate power to examine the effect of child sex and other factors that may influence our results. Furthermore, prenatal medication use may affect DNAm patterns [52], but we did not obtain information on medications that mothers may have been taking during their pregnancies. Therefore, future studies are needed to replicate the findings from our study using larger cohorts, more detailed maternal assessments, and biochemical verification of prenatal smoke exposure.

In conclusion, results from this study provide further support that early life exposures are associated with changes in the epigenome. Early life exposure to smoking is a complex and persistent public health problem in the United States and globally. Due to the mounting evidence that prenatal tobacco smoke exposure leads to global poor health outcomes, prevention efforts are urgently needed. Future studies are needed to identify how gene expression in response to early environmental exposures contributes to health outcomes, including how gene expression of *AVPR1a* contributes to cardiovascular outcomes, such as high blood pressure. Results from such studies have the potential to lead to screening and prevention services among high-risk populations, such as mother–child dyads enrolled in home visiting with the goal of improving maternal and child health outcomes.

## Figures and Tables

**Table 1:** Study Population Characteristics.

	Non-Smoker (N = 42)	Smoker (N = 10)	*p* Value
	N (%)	N (%)	
**Age**			0.3
18–20	20 (47.6)	2 (20.0)	
21–24	17 (40.5)	6 (60.0)	
25–34	5 (11.9)	2 (20.0)	
**Substance Use**			0.04
No	40 (95.2)	7 (70.0)	
Yes	2 (4.8)	3 (30.0)	
**Race**			
Non-White	30 (71.4)	5 (50.0)	0.26
White	12 (28.6)	5 (50.0)	
**Low Income**			
No	1 (2.6)	0 (0.0)	1
Yes	38 (97.4)	10 (100.0)	
**Education**			0.5
Bachelor’s Degree	1(2.4)	0 (0)	
GED	2 (4.8)	2 (20.0)	
High School Diploma	27 (64.3)	6 (60.0)	
Some College or 2 yr. degree	8 (19.1)	1 (10.0)	
Some High School	2 (4.8)	1 (10.0)	
Technical or Trade School	2 (4.8)	0 (0.0)	
**Breastfed**			0.05
No	3 (7.3)	3 (37.5)	
Yes	38 (92.7)	5 (62.5)	
**Infant Sex**			
Female	22 (52.4)	6 (60.0)	0.74
Male	20 (47.6)	4 (40.0)	
**Preterm Birth**			0.02
No	39 (92.9)	6 (60.0)	
Yes	3 (7.1)	4 (40.0)	
**Medicaid**			1
No	2 (5.0)	0 (0.0)	
Yes	38 (95.0)	10 (100.0)	

Abbreviations: GED—General Educational Diploma.

**Table 2:** DNAm (beta-value) of CpG sites within *AVPR1a* and Association with Maternal Smoking.

Maternal Smoking DNAm by CpG Site	Adjusted Model Coefficient * (95% CI)	*p* Value **	Bonferroni	FDR	Location	CPG	Enhancer
cpg12807275	−0.12 (−0.19, −0.05)	0.00	0.01	0.01	Body	N_Shore	No
cpg04827692	−0.04 (−0.06, −0.01)	0.01	0.03	0.01	1stExon	Island	No
cpg16668728	−0.00 (−0.01, 0.01)	0.72	1.00	0.81	1stExon	Island	Yes
cpg16352140	−0.00 (−0.01, 0.01)	0.40	1.00	0.53	1stExon	Island	Yes
cpg24501701	−0.00 (−0.02, 0.01)	0.81	1.00	0.81	1stExon	Island	Yes
cpg26727693	−0.01 (−0.02, 0.01)	0.29	1.00	0.47	1stExon	Island	Yes
cpg09208611	−0.01 (−0.02, −0.00)	0.00	0.03	0.01	1stExon	Island	Yes
cpg10906284	−0.00 (−0.01, 0.00)	0.13	0.65	0.26	1stExon	Island	Yes

Abbreviations: DNAm–DNA methylation; CpG-cytosine-phosphate-guanine dinucleotides: FDR—false discovery rate. Model adjusted for substance use, * DNAm beta-value as the outcome, ** from model with DNAm m-value as the outcome.

## Data Availability

The data presented in this study are available on request from the corresponding author. The data are not publicly available due to privacy restrictions.
